# High-quality chromosome-level genome assembly of the Northern Pacific sea star *Asterias amurensis*

**DOI:** 10.1093/dnares/dsae007

**Published:** 2024-02-28

**Authors:** Zhichao Huang, Qi Liu, Xiaoqi Zeng, Gang Ni

**Affiliations:** Ministry of Education Key Laboratory of Mariculture, Ocean University of China, Qingdao 266003, China; Wuhan Onemore-tech Co., Ltd, Wuhan 430000, China; Ministry of Education Key Laboratory of Mariculture, Ocean University of China, Qingdao 266003, China; Institute of Evolution and Marine Biodiversity, Ocean University of China, Qingdao 266003, China; Ministry of Education Key Laboratory of Mariculture, Ocean University of China, Qingdao 266003, China

**Keywords:** *Asterias amurensis*, genome assembly, Hi-C, comparative genomics

## Abstract

*Asterias amurensis*, a starfish species that is native to countries such as China and Japan, as well as non-native regions like Australia, has raised serious concerns in terms of its impact on ecology and economy. To gain a better understanding of its population genomics and dynamics, we successfully assembled a high-quality chromosome-level genome of *A. amurensis* using PacBio and Hi-C sequencing technologies. A total of 87 scaffolds assembly with contig N50 length of 10.85 Mb and scaffold N50 length of 23.34 Mb were obtained, with over 98.80% (0.48 Gb) of them anchored to 22 pseudochromosomes. We predicted 16,673 protein-coding genes, 95.19% of which were functionally annotated. Our phylogenetic analysis revealed that *A. amurensis* and *Asterias rubens* formed a clade, and their divergence time was estimated ~ 28 million years ago (Mya). The significantly enriched pathways and Gene Ontology terms related to the amplified gene family were mainly associated with immune response and energy metabolism, suggesting that these factors might have contributed to the adaptability of *A. amurensis* to its environment. This study provides valuable genomic resources for comprehending the genetics, dynamics, and evolution of *A. amurensis*, especially when population outbreaks or invasions occur.

## 1. Introduction

Starfish are voracious predators with multiple arms that feed on commercial shellfish such as oysters, scallops, clams, and other bivalves, as well as live corals.^1–3^ The characteristics and dietary habits of starfish ensure their rapid proliferation when environmental conditions are favourable, leading to massive outbreaks known as starfish disasters, which can cause significant damage to shellfish farming and coral ecosystems.^4–6^ The outbreak of starfish along coastal areas will result in severe economic losses and ecological damage to the aquaculture industry.

*Asterias amurensis* (Asteriidae, Forcipulatida) was originally distributed in the distant North Pacific region,^7^ encompassing North China, Japan, Russia, and Korea. As an invasive species, it has successfully established in southern Australia^8^ and has become one of the most severe invasive marine pests here.^9^ This starfish lives in a variety of marine habitats where it feeds on various prey (e.g. bivalves including oysters, scallops, and clams) that may dramatically alter community structure.^10^ Adult *A. amurensis* has few natural enemies and exhibits strong reproductive ability,^1^ ensuring its sudden population proliferate when environmental conditions are favourable.^11^ Over the past decades, outbreaks of this starfish had been reported in coastal areas of China, Japan, South Korea, and Australia,^12–14^ which had caused significant losses to local shellfish fisheries and coastal ecosystems.^15^ However, starfish of this genus receive much less attention compared to crown-of-thorns starfish, which feed on precious coral reefs. Molecular biology research focussing on the genetic diversity of *A. amurensis* has been relatively limited, but see Matsubara et al.^16^ So far, studies on the genetic diversity of the *A. amurensis* have been limited to the analysis of mitochondrial CO1 and microsatellite markers, without delving into the analysis at the whole-genome level.^17^

The use of genomic information holds great promise in understanding and monitoring population structure and dynamics.^18,19^ High-quality genome assembly sequences enable comprehensive and scientific decoding of genetic diversity in various organisms.^20^ They have been extensively utilized to study invasion dynamics, identify molecular mechanisms underlying adaptability, and discover promising genes for biotechnology-based control strategies.^21^ To fill the gap, in this study, we successfully assembled a high-quality chromosome-level genome of *A. amurensis* using Illumina, PacBio and Hi-C sequencing technologies resulting in 22 pseudochromosomes. We then conducted a comparative analysis of the *A. amurensis’* genome with genomes of seven other Echinoderms, including Crinoidea, Echinoidea, and Asteroidea. These analyses provide valuable insights into the evolution of starfish and the genetic basis of their environmental adaptability.

## 2. Materials and methods

### 2.1. Sample collection

An adult female seastar was caught by hand at low tide from Dagong Island, Qingdao, China (N35°57ʹ36.5″, E120°29ʹ31.8″) in August 2021. Tissues including tube feet, muscle, gonad, pyloric stomach, and digestive gland were collected, immediately frozen in liquid nitrogen and stored at −80°C before DNA extraction. Genomic DNA was extracted from muscle tissue using the standard phenol-chloroform method, and the quality and concentration were assessed through 1% agarose gel electrophoresis and the Pultton DNA/Protein Analyzer (Plextech). Total RNA was extracted from each tissue above by using TRIzol reagent (Invitrogen), and its quality was determined using a Qubit fluorometer and Nanodrop spectrophotometer (Labtech).

### 2.2. Genome sequencing

The Illumina NovaSeq 6000 and PacBio Sequel II platforms were applied for genomic sequencing to generate short and long genomic reads, respectively. Paired-end libraries were constructed with an insert size of 300–350 bp according to the standard Illumina protocols. For long-read sequencing, we constructed a Single Molecule Real-Time (SMRT) bell library with a fragment size of 20 kb following the manufacturer’s protocol. The library was sequenced with one SMRT cell, which was mainly used to assemble the whole genome. To obtain a chromosome-level genome assembly, a Hi-C library was prepared following Hi-C library protocols and sequenced using the Illumina Novaseq 6000 sequencing platform.^22^

### 2.3. Genome assembly and assembly evaluation

The *K*-mer based method of the Illumina short-read data was used to analyse the genome survey with GCE (v1.0.0) to estimate the genome size, heterozygosity, and repeat content, in which the *K* = 17.^23,24^ A SMRT genome sequencing library was constructed using the PacBio Sequel II platform.^25,26^ The PacBio long reads were used for *de novo* genome assembly with HiFiasm (v0.16.1-r375).^27^ De-redundancy of the assembled genome’s initial assembly and error correction were performed using Purge_haplotigs (v1.0.4).^28^

The integrity of the *A. amurensis* genome was evaluated using Benchmarking Universal Single-Copy Orthologs (BUSCO v5.3.1).^29^ In order to confirm the assembly results belonging to the target species, the genome sequence was fragmented into segments of 1,000 bp using the software Blast and aligned against the NCBI nucleotide (NT) database.^30^  Supplementary Table 1 presents the top five genera ranked by the number of alignments. Genome assembly quality control was used for the distribution of Guanine/Cytosine (GC) depth. Finally, the gene density, repeat density, and GC density distributions of the assembled genome of *A. amurensis* were calculated and plotted as scatter plots.

### 2.4. Chromosome assembly

After the genome has undergone assisted assembly, Juicer is utilized to construct an interactome map, and JuiceBox is employed for visual error correction.^31,32^

### 2.5. Genome annotation

Genome annotation mainly includes three aspects: repetitive recognition, non-coding RNA prediction, gene structure prediction, and functional annotation.

Homology prediction using RepeatMasker (vopen-4.0.9) and RepeatProteinMask based on RepBase (http://www.girinst.org/repbase) and *de novo* prediction using RepeatModeler (v open-1.0.11), Piler, RepeatScount based on Self-sequence alignment and using Trf (v4.09) and LTR-FINDER based on repeat sequence feature were combined to annotate the repetitive sequences of *A. amurensis* genome.^33–36^

Combined with homologous prediction, *de novo* prediction (software: Augustus, Genscan, GlimmerHMM), cDNA/EST prediction to make structural prediction of coding genes.^37,38^ Meanwhile, RNA-seq data (accession numbers: SRR26104401, BioProject ID: PRJNA1016059) were compiled by Tophat alignment and Cufflinks assembled transcripts.^39^ By using the MAKER, the predicted gene sets can be integrated into a non-redundant and more comprehensive gene set. Additionally, by incorporating the CEGMA results and implementing the HiCESAP workflow, a final reliable gene set can be obtained.^40^ Finally, the proteins in the gene set will be functionally annotated using external protein databases such as SwissProt, TrEMBL, Kyoto Encyclopedia of Genes and Genomes (KEGG), InterPro, and Gene Ontology (GO).^41,42^

The tRNAscan-SE software was used to find tRNA sequences in the genome. The reference sequence for rRNA from Invertebrates is selected, and BLASTN alignment is performed to identify rRNA sequences within the genome. By utilizing the covariance models from the Rfam database and employing the INFERNAL software provided by Rfam, it is possible to predict the miRNA and snRNA sequences present in the genome.^43,44^

### 2.6. Gene families and phylogenetic tree construction

To gain a deeper understanding of the evolutionary gene family in Echinoderm, we compared the genes families of *A. amurensis*^45^ with the genomes of the following Echinoderm: *Acanthaster planci* (accession number: GCF_001949145.1), *Anneissia japonica* (accession number: PRJNA553656), *Asterias rubens* (accession number: GCF_902459465.1), *Lytechinus pictus* (accession number: GCF_015342785.2), *Lytechinus variegatus* (accession number: GCF_018143015.1), *Patiria miniata* (accession number: GCF_015706575.1), and *Strongylocentrotus purpuratus* (accession number: GCF_000002235.5).

When multiple transcripts (alternative splicing) exist for a gene, only the transcript with the longest coding region remains. Genes encoding proteins of less than 30 amino acids or genes with stop codons inside are filtered out. The similarity relationship between all species protein sequences was obtained by all-vs-all blastp, and 1e−5 was used for e-values. The above results were clustered using OrthoMCL with a coefficient of expansion of 1.5.^46^

Genes from single-copy gene families are selected for further analysis. The obtained single-copy orthologous genes were aligned using MAFFT (v7.487),^47,48^ and the resulting alignment was converted into a multiple sequence alignment of coding sequences (CDS). The alignments of all single-copy genes were merged to construct a super alignment matrix. Finally, a phylogenetic tree was constructed using the maximum likelihood (ML) method in RAxML (v8.2.12).^48^ Based on gene sequences from single-copy gene families, divergence time estimation was performed using the PAML MCMCTree.^49^ Several key node times used for correction were found at TimeTree (http://www.timetree.org/).

### 2.7. Gene families expansion

Based on gene family evolutionary models, CAFE was used to calculate the *P* values associated with the gene family size correlation between *A. amurensis* and the crown-of-thorns starfish *A. planci* with extant species.^50^ The genes were functionally classified based on the GO annotation results and official classification. The clusterProfiler function in *R* was used for enrichment analysis, and the *P*-value was calculated. Similarly, based on the KEGG annotation results and official classification, the genes were categorized into biological pathways, and the clusterProfiler function in *R* was used for enrichment analysis, with *P*-value being calculated. The KEGG and GO enrichment results were compared between *A. amurensis* and the crown-of-thorns starfish *A. planci.*

### 2.8. Collinearity analysis

*Asterias rubens* which is closely related to *A. amurensis* in the genus was selected for interspecific collinearity analysis. Collinearly analyses were performed based on both coding genes and whole genomes, using JCVI and Mumme softwares, respectively.^51^

## 3. Result and discussion

### 3.1. Assembly of a high-quality *A. amurensis* genome

**Figure 1.**
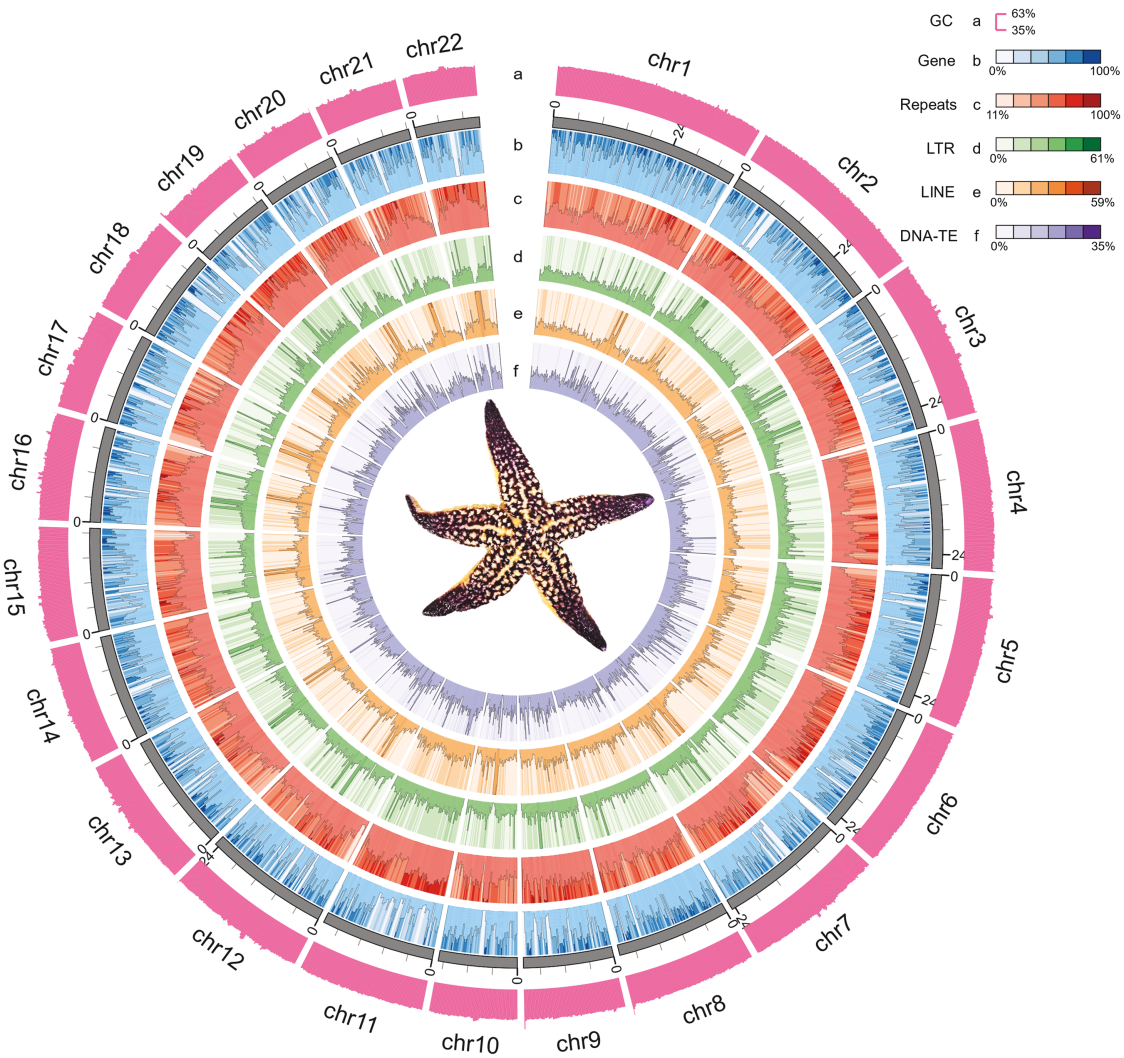
Circular map of *A. amurensis* genome. a: GC content, b: gene density, c: all repeats density distributions except TRF, d: LTR type repeat density distribution, e: LINE type repeat density distribution, f: DNA-TE type repeat density distribution.

A total of 79.91 Gb of clean data was acquired using the Illumina NovaSeq 6000 platform. *K*-mer analysis showed that the sample genome size was 484 Mb after correction with a heterozygosity rate of 0.96% and repeat sequence ratio of 39.22%. Using the PacBio Sequel II platform, 25.97 Gb of PacBio HiFi circular consensus sequencing (CCS) reads were obtained. A total of 87.30 Gb of clean Hi-C reads were generated, and 0.48 Gb (98.80%) of the long-read genome assembly was anchored to 22 pseudochromosomes. The 22 pseudochromosomes were clearly distinguished from the Hi-C heatmap and interactions within the pseudochromosomes were strong (Supplementary Fig. S2), indicating a high-quality anchoring. The final assembly yielded 22 chromosomes and 87 scaffolds with a total length of 0.48 Gb, with contig N50 of 10.85 Mb and scaffold N50 of 23.34 Mb (Table 1). The distributions of gene density, GC content, and repeating density of the 22 pseudochromosomes are shown in Fig. 1.

**Table 1.** Statistics of *A. amurensis* genome assembly

Assembly statistics	Value
Genome size (bp)	482,994,179
Number of scaffolds	87
Number of chromosome-scale scaffolds	22
Contig N50 (bp)	10,846,023
Scaffold N50 (bp)	23,343,777
GC content (%)	39.09

By comparing against the metazoa_odb10 database, the BUSCO analysis revealed that 98.4% complete BUSCOs, 97.7% of which were complete and single copies and 0.7% of which were complete and duplicated (Table 2). In total, 49,560 genomic fragments (based on a step length of 1 kb) were randomly selected and mapped to the NCBI Nucleotide (NT) database, with more than 99.97% of these fragments aligned to *Asterias* genomes (Supplementary Table S1). The GC_depth scatter plots demonstrated a Poisson distribution, indicating that this genome had no exogenous contamination (Supplementary Fig. S1). Based on the evaluation of the genome assembly, the next-generation reads were aligned to the genome. Subsequently, the software Samtools was used for sorting, Picard for duplicate removal, and GATK for variant detection.^52–54^ We obtained 0.632% heterozygous single nucleotide polymorphisms (SNP) and 0.07% homozygous SNPs. In addition, the homozygous and heterozygous insertion–deletion (InDel) rates were 0.001% and 0.194%, respectively. These results indicated a high degree of integrity in genome assembly.

**Table 2.** Genome assembly and annotation evaluation

	Genome assembly		Genome annotation	
	Proteins	Percentage (%)	Proteins	Percentage (%)
Complete	939	98.4	917	96.1
Complete and single-copy	932	97.7	909	95.3
Complete and duplicated	7	0.7	8	0.8
Fragmented	4	0.4	11	1.2
Missing	11	1.2	26	2.7
Total	954	100	954	100

### 3.2. Genome annotation

A total of 300.45 Mb of repeat sequences were detected, accounting for 61.64% of the assembled genome (Supplementary Table S2), as predicted by TRF (13.95%), RepeatMasker (3.18%), RepeatProteinMask (2.06%), and *de novo* (56.91%). Repetitive sequences primarily consisted of mainly long terminal repeats (LTRs, 19.80%), followed by long interspersed nuclear elements (LINEs, 18.18%), DNA transposons (14.31%), short interspersed nuclear elements (SINEs, 13.74%), and satellite (1.80%) (Supplementary Fig. S3 and Table S3). Approximately, 12.55% of the genome was annotated as unknown repetitive sequences.

Homology Prediction and *de novo* prediction were used in combination for gene prediction of the genome, predicting 16,673 protein-coding genes. Average gene length, average CDS length, average exon length, average intron length, and average exon number per gene were 16,777.57 bp, 1,717.80 bp, 385.28 bp, 1,664.55 bp, and 8.99, respectively (Supplementary Table S4). A total of 15,871 genes, accounting for 95.19% of all predicted genes, were annotated using public databases (Supplementary Table S5).

For non-coding RNA predictions, we successfully annotated 36 microRNAs (miRNAs), 6779 transfer RNAs (tRNAs), 864 ribosomal RNAs (rRNAs), and 171 small nuclear RNAs (snRNAs), with average lengths of 88 bp, 74 bp, 469 bp, and 171 bp, respectively (Supplementary Table S6).

### 3.3. Genome annotation evaluation

BUSCO was also used to test the completeness of the genome annotation with the metazoa_odb10 database, which showed that 909 complete single-copy BUSCOs and eight complete duplicated BUSCOs (Table 2) were predicted for *A. amurensis*.

### 3.4. Phylogenetic analysis and syntenic relationship

**Figure 2.**
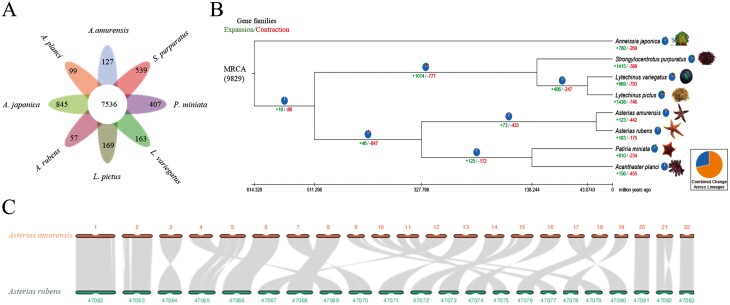
Phylogenetic relationship and comparative genomics analyses. (A) Venn diagram showing the orthologous gene families shared among the genomes of *A. amurensis*, *Acanthaster planci*, *Anneissia japonica*, *Asterias rubens*, *Lytechinus pictus*, *Lytechinus variegatus*, *Patiria miniata*, and *Strongylocentrotus purpuratus* (B) A phylogenetic tree of *A. amurensis* and seven other species. The numbers of gene families that expanded or contracted in each lineage after speciation are shown in the circles of the corresponding branch. (C) Gene comparison of homologous chromosomes between *A. amurensis* and *A. rubens*. Grey lines indicate collinearity between the genomes.

To investigate the genomic evolution of Echinoderms, we compared the genome sequences of eight species (*A. amurensis*, *A. planci*, *A. japonica*, *A. rubens*, *L. pictus*, *L. variegatus*, *P. miniata*, and *S. purpuratus*) of Echinoderms and clustered these genes into 7,536 gene families (Fig. 2A). A total of 2,812 single-copy gene families were identified and used to construct a phylogenetic tree. According to phylogenetic analysis, the divergence time between the class Asteroidea and other Echinoderms is estimated to be approximately 511.298 (461.1–600.0) million years ago (Mya) (Fig. 2B). *Asterias amurensis* and *A. rubens* formed a clade, and their divergence time was estimated ~ 28 Mya. The class Crinoidea represents the most distant lineage from Asteroidea in terms of their phylogenetic relationship. The above results provide support for the reliability of the phylogenetic tree.

Collinearity analysis enables researchers to assess evolutionary events at the molecular level between different species, as well as explain the structural variations observed between two genomes. The chromosomes of both *A. amurensis* and *A. rubens* exhibit remarkable interchromosomal matching, with nearly every chromosome demonstrating a high degree of shared characteristics, showcasing an exceptional level of conserved genomic traits (Fig. 2C). This indicates that they have a close phylogenetic relationship and is consistent with the results of the phylogenetic tree analysis.

### 3.5. Expansion of gene families

**Figure 3.**
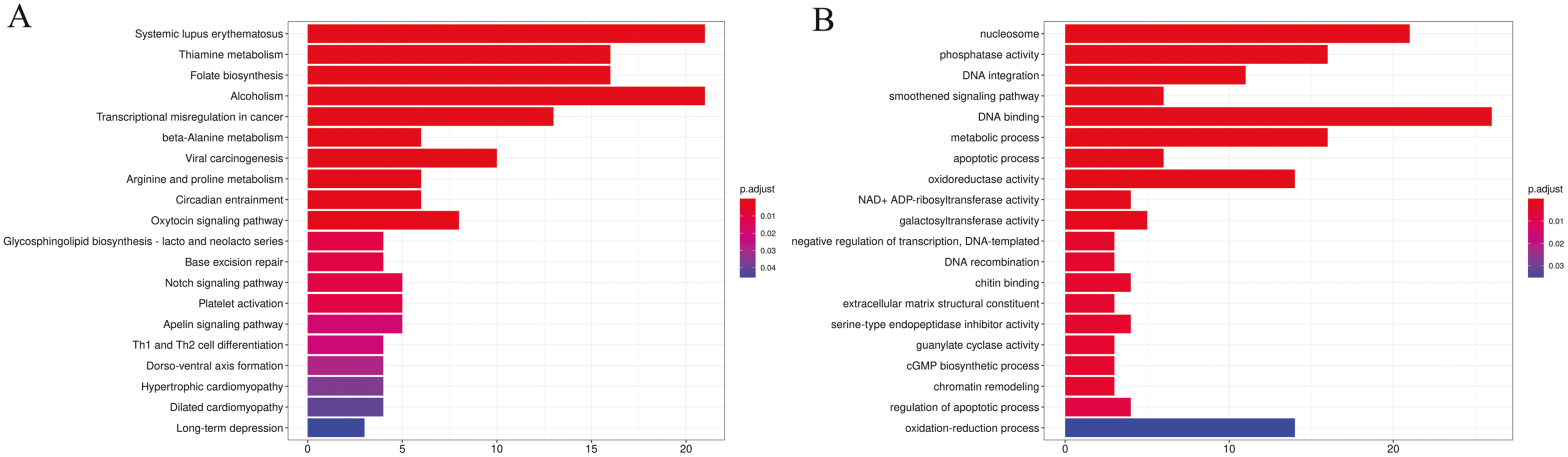
(A) KEGG pathway enrichment analysis of significantly expanded gene families. The horizontal axis represents the number of genes, the vertical axis represents the KEGG Pathway, and the colour indicates the *q*-value. A smaller *q*-value indicates a more significant enrichment result. (B) GO enrichment analysis of significantly expanded gene families. The horizontal axis represents the number of genes, the vertical axis represents the Gene Ontology functional categories, and the colour indicates the *q*-value. A smaller *q*-value indicates a more significant enrichment result.

The KEGG and GO enrichment results were shown in Fig. 3A and B. Significant pathway amplifications (Supplementary Table S7) were observed in immune response, energy metabolism, and signal transduction functions, including the beta-alanine metabolism (ko00410, six genes, *P* = 1.22E−05), arginine and proline metabolism (ko00330, six genes, *P* = 0.00017), glycosphingolipid biosynthesis-lacto and neolacto series (ko00601, four genes, *P* = 0.00230), and Th1 and Th2 cell differentiation (ko04658, four genes, *P* = 0.00596).

The 26 significantly enriched GO terms (Supplementary Table S8), including phosphatase activity (GO:0016791, 16 genes, *P* = 6.00E−13), metabolic process (GO:0008152, 16 genes, *P* = 8.35E−07), oxidoreductase activity (GO:0016491, 14 genes, *P* = 2.14E−05), guanylate cyclase activity (GO:0004383, three genes, *P* = 0.00161), and oxidation–reduction process (GO:0055114, 14 genes, *P* = 0.01312), were related to the functions of cell signalling, metabolism, and regulation.

Former studies have proved that these pathways and gene families with significant expansion could potentially influence various physiological processes and enhance species adaptability to the environment.^55–58^ In *A. amurensis*, specifically, we speculated that under stress conditions, the expansion of the beta-Alanine metabolism pathway may be involved in regulating energy metabolism, antioxidant reactions, or other adaptive mechanisms. The expansion of the arginine and proline metabolism pathway is likely an adaptive response of *A. amurensis* to environmental or internal stimuli. These metabolic pathways may play a role in regulating cellular nitrogen balance, stress response, immune regulation, and other physiological processes. The expansion of Th1 and Th2 cells, as distinct immune system subgroups serving crucial regulatory functions in orchestrating immune responses, suggests that the immune system of *A. amurensis* exhibits enhanced adaptability to both cell-mediated immunity and humoral immunity.

After compared the results above with the KEGG and GO enrichment results of gene families in the crown-of-thorns starfish *A. planci* (Supplementary Tables S9 and S10), we found that: In the KEGG enrichment analysis, gene families of Base Excision Repair, Cell Cycle, Oocyte Meiosis, and Apoptosis were significantly enriched in both species; In GO enrichment analysis, significant enrichment of carbohydrate metabolic process, DNA repair, DNA integration, and regulation of apoptotic process gene families in both species.

## 4. Conclusion

In the present study, we assembled the chromosome-level genome of *A. amurensis* and performed relevant annotations. After utilizing Hi-C technology for genome assembly, the entire genomic sequence was successfully anchored to 22 chromosomes, achieving an anchoring rate of 98.80%. The genomes of *A. amurensis* and its congeneric species, *A. rubens*, exhibit a high degree of conservation. This opens the door for comparative studies at the genomic level and provides insights into the evolution of *A. amurensis* and other genomes. This study offers valuable genomic data for further exploring the molecular mechanisms underlying the biological characteristics and functional validation of candidate genes of *A. amurensis* and provides valuable insights into the molecular evolution of *A. amurensis* and other starfish, serving as a reliable reference for future sequencing studies. In addition, at the population genomic level, by assessing the genomic variations among native and invasive populations, we can uncover potential pathways of dispersal between them and contribute to the development of more effective control policies.

## Supplementary Material

dsae007_suppl_Supplementary_Data

## Data Availability

The raw sequencing data for the *A. amurensis* genome, including Illumina, PacBio, Hi-C, and RNA-seq reads, have been deposited at the National Center for Biotechnology Information (NCBI) sequence read archive (SRA). The accession numbers for these datasets are SRR26104404, SRR26104403, SRR26104402, and SRR26104401. They are associated with the BioProjectID PRJNA1017625. Genomic raw sequencing data were also archived in the Science Data Bank database (https://www.scidb.cn/s/7BZBre). The assembled genome archived in the Figshare with the URL as follows: https://doi.org/10.6084/m9.figshare.24708021.v2.
