# Supplementary material for: High-quality chromosome-level genome assembly of the Northern Pacific sea star Asterias amurensis
Source: DNA Res. 2024 Feb 28;31(2):dsae007. doi: 10.1093/dnares/dsae007 (PMC11090083; doi:10.1093/dnares/dsae007)
Supplement: dsae007_suppl_Supplementary_Data [file dsae007_suppl_supplementary_data.zip › Supplementary_file/Supplementary Data.docx]

Supplementary data are available at DNARES online.

**Table S1:** *Asterias amurensis* sequence alignment with NT database

**Table S2:** Annotated repeat sequences in *A. amurensis* genome

**Table S3:** Transposable elements in *A. amurensis* genome

**Table S4:** Gene predictions in *A. amurensis* genome

**Table S5:** Functional annotations of predicted genes

**Table S6:** Non-coding RNA annotation

**Table S7**: KEGG enrichment analysis of significantly expanded gene families in *A. amurensis*

**Table S8:** GO enrichment analysis of significantly expanded gene families in *A. amurensis*

**Table S9**: KEGG enrichment analysis of significantly expanded gene families in the crown-of-thorns starfish

**Table S10:** GO enrichment analysis of significantly expanded gene families in the crown-of-thorns starfish

**Figure S1:** A: Statistical chart of correlation analysis between GC content and coverage depth (second-generation reads comparison). B: Statistical chart of correlation analysis between GC content and coverage depth (third-generation reads comparison).

**Figure S2:** Heatmap of chromosome interaction intensity in *Asterias amurensis* Hi-C assembly.

**Figure S3:** RepeatableModeler transposon differentiation rate. The abscissa is the divergence degree between annotated TE sequences in the genome and corresponding sequences in the total library file, and the ordinate is the percentage of TE sequences under the divergence degree in the genome. Different TEs are labeled in different colors: DNA-red, LINE-black, LTR- yellow, and SINE-green.

**Figure S4:** Statistics of the number of homologous genes in species.
